# Supplementary material for: No Difference in the Prevalence of HIV-1 gag Cytotoxic T-Lymphocyte-Associated Escape Mutations in Viral Sequences from Early and Late Parts of the HIV-1 Subtype C Pandemic in Botswana
Source: Vaccines (Basel). 2023 May 19;11(5):1000. doi: 10.3390/vaccines11051000 (PMC10221913; doi:10.3390/vaccines11051000)
Supplement: Supplementary file 1 [file vaccines-11-01000-s001.zip › vaccines-2390797-supplementary.pdf]

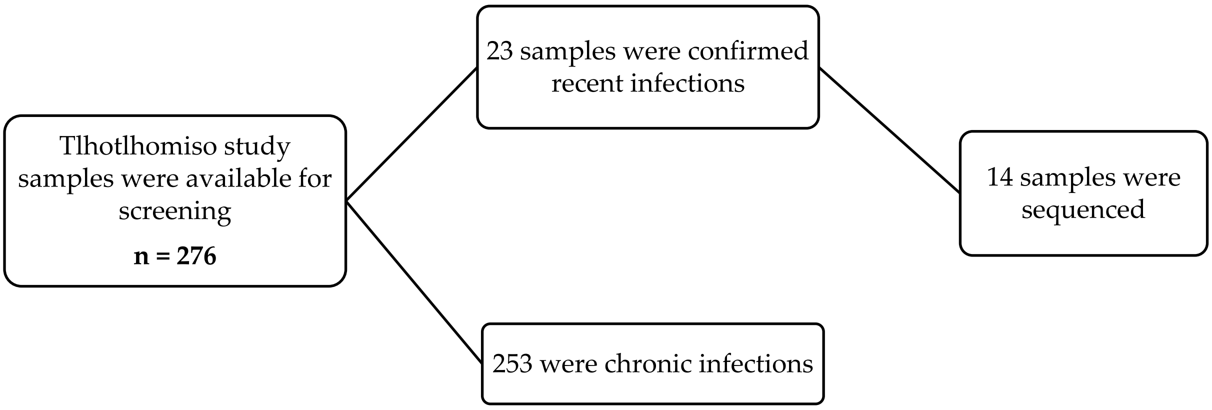

Supplementary Figure S1. The selection of 14 recent infections from Tlhotlhomiso study used the LTP.

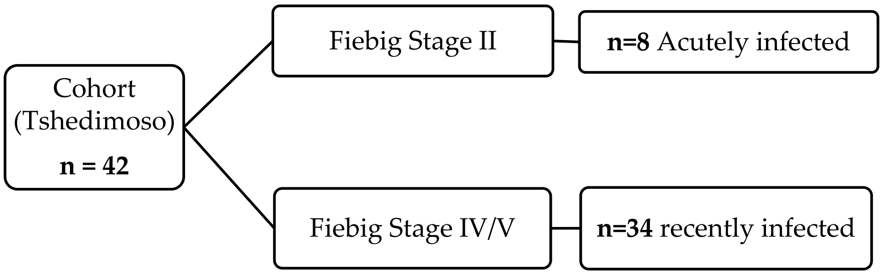

Supplementary Figure S2. The selection of 42 sequences used as early time point (Tshedimoso study).

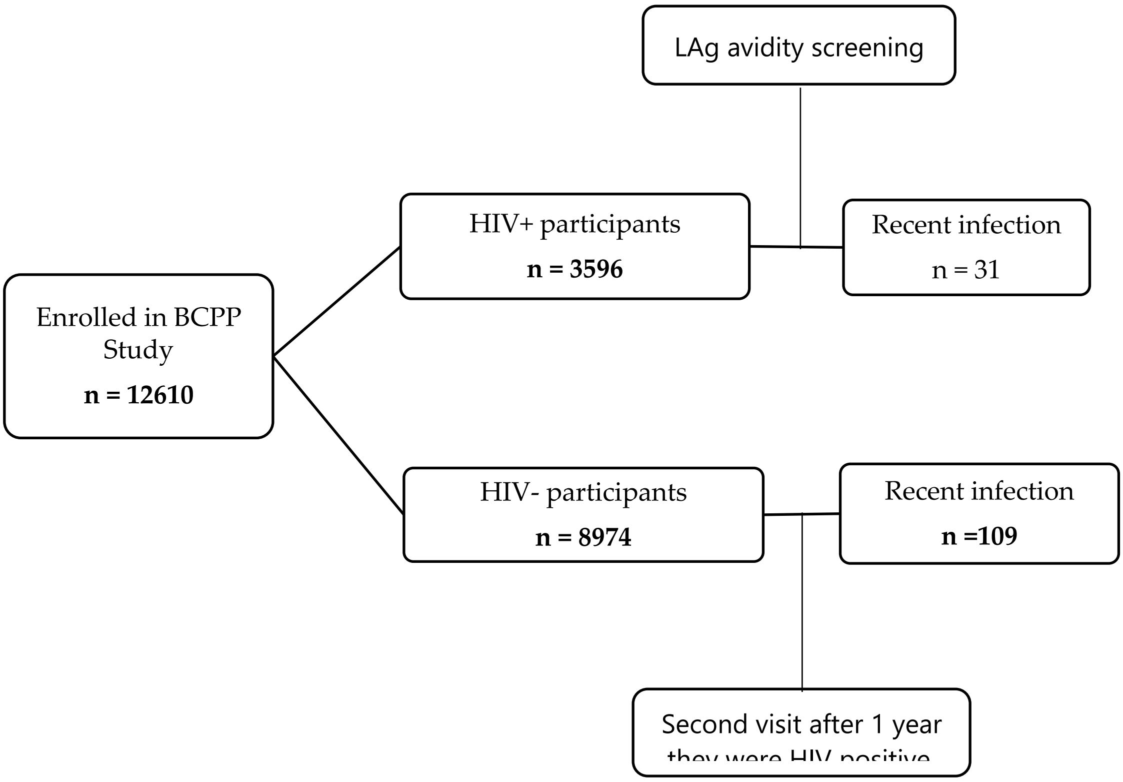

Supplementary Figure S3. The selection of 140 BCPP study for recent infection used LTP.

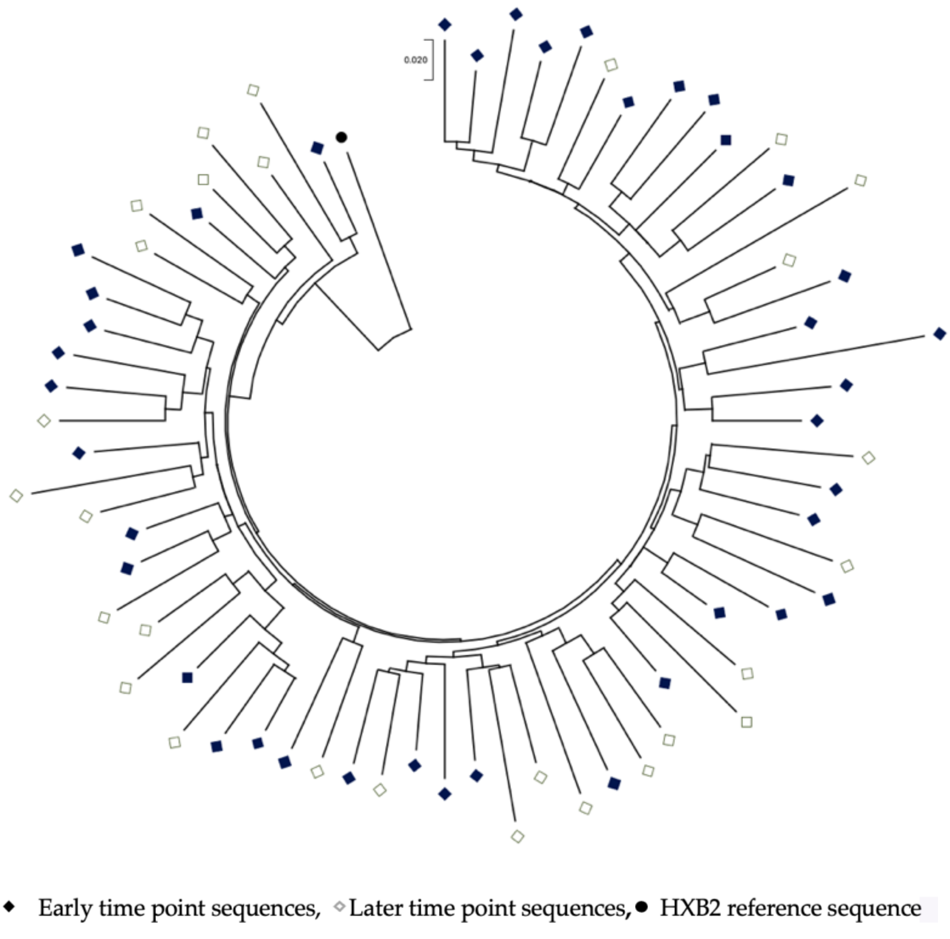

Supplementary Figure S4. Maximum likelihood phylogenetic tree based on p17 (aa = 130 base-pairs).

Supplementary Table S1: Deleterious and neutral escape mutations in HIV-1C *gag* that have not been reported.

HIV-1C variant	*PROVEAN SCORE*	*PROVEAN prediction*
K26G	-3.833	Deleterious
E52L	-4.933	Deleterious
E55A	-3.348	Deleterious
I60Q	-4.049	Deleterious
L64Q	-3.711	Deleterious
L75E	-3.945	Deleterious
V88C	-3.020	Deleterious
T97D	-3.651	Deleterious
Q108E	-1.484	Neutral
S111K	-1.784	Neutral
K113C	-3.050	Deleterious
K114Q	-1.699	Neutral
Q116T	-2.800	Deleterious
A118Q	-2.181	Neutral
A120E	-1.465	neutral
A120K	-1.626	Neutral
D121A	0.458	Neutral
T122A	1.412	Neutral
T122D	0.127	Neutral
G123D	-2.543	Deleterious
H124D	0.127	Neutral
S125K	-1.157	Neutral
V159I	0.043	Neutral
S173T	0.627	Neutral
E203D	-3.455	Deleterious
T375N	0.890	Neutral
T375S	0.123	Neutral

The prevalence of these mutations was higher at ETP compared to LTP, all the p-values were <0.01.
